# Comparative proteomic profiling of the ovine and human PBMC inflammatory response

**DOI:** 10.1038/s41598-024-66059-0

**Published:** 2024-06-28

**Authors:** A. Elkhamary, I. Gerner, A. Bileck, G. L. Oreff, C. Gerner, F. Jenner

**Affiliations:** 1 Department for Companion Animals and Horses, Veterm, University Equine Hospital, Vetmeduni Vienna, Vienna, Austria; 2 Department for Surgery, Faculty of Veterinary Medicine, Damanhour University, Damanhour, Egypt; 3 Austrian Cluster for Tissue Regeneration, Vienna, Austria; 4 Department of Analytical Chemistry, Faculty of Chemistry, University of Vienna, Vienna, Austria

**Keywords:** Experimental models of disease, Inflammation

## Abstract

Understanding the cellular and molecular mechanisms of inflammation requires robust animal models. Sheep are commonly used in immune-related studies, yet the validity of sheep as animal models for immune and inflammatory diseases remains to be established. This cross-species comparative study analyzed the in vitro inflammatory response of ovine (oPBMCs) and human PBMCs (hPBMCs) using mass spectrometry, profiling the proteome of the secretome and whole cell lysate. Of the entire cell lysate proteome (oPBMCs: 4217, hPBMCs: 4574 proteins) 47.8% and in the secretome proteome (oPBMCs: 1913, hPBMCs: 1375 proteins) 32.8% were orthologous between species, among them 32 orthologous CD antigens, indicating the presence of six immune cell subsets. Following inflammatory stimulation, 71 proteins in oPBMCs and 176 in hPBMCs showed differential abundance, with only 7 overlapping. Network and Gene Ontology analyses identified 16 shared inflammatory-related terms and 17 canonical pathways with similar activation/inhibition patterns in both species, demonstrating significant conservation in specific immune and inflammatory responses. However, ovine PMBCs also contained a unique WC1^+^γδ T-cell subset, not detected in hPBMCs. Furthermore, differences in the activation/inhibition trends of seven canonical pathways and the sets of DAPs between sheep and humans, emphasize the need to consider interspecies differences in translational studies and inflammation research.

## Introduction

Inflammation is a double-edged sword in maintaining health and disease, integral to both the physiologic response to injury or infection and, when chronic, the pathogenesis of most chronic diseases, including cardiovascular disease, diabetes, osteoarthritis and cancer in both humans and animals^1–9^. Indeed, chronic inflammatory diseases globally account for more than 50% of all deaths, making them the most significant cause of morbidity and mortality^2,3^.

Inflammation is an evolutionarily conserved protective response to external and internal injurious stimuli, such as invading pathogens, toxins or damaged cells. It serves to eliminate the noxae, clear necrotic cells, initiate tissue repair and restore tissue homeostasis^4,6–15^. The inflammatory response starts when tissuE-resident sentinel cells’ pattern recognition receptors detect pathogen- or damage-associated molecular patterns. This triggers the secretion of inflammatory cytokines, leading to the recruitment of neutrophils and peripheral blood mononuclear cells (PBMCs) for phagocytosis and elimination of tissue debris and microorganisms^4,6–15^. Recruited leukocytes, activated by the local inflammatory environment, adopt an inflammatory phenotype, secreting proteases, chemokines, and cytokines, amplifying inflammation^16–18^. However, acute inflammation is typically short-lived and subsides once the trigger is eliminated^18–34^. Thereafter, macrophages clear apoptotic neutrophils through efferocytosis, initiating their functional repolarization to a pro-resolving phenotype and biosynthesis of pro-resolving mediators that promote the return to homeostasis^18–34^. Thus, the delicate balance between an effective defensive response, collateral tissue damage and persistent inflammation hinges on the tightly coordinated regulation of pro-inflammatory and pro-resolving cytokine secretion^18–34^. However, the intricate signaling cascades orchestrating an inflammatory response and its resolution or transition to chronic inflammation remain to be fully elucidated.

Moreover, during both systemic inflammatory responses and localized inflammatory reactions, cytokines, such as interleukin (IL)-6 and tumor necrosis factor (TNF)-α, are released into the bloodstream, orchestrating a range of circulating immune cell activities, including activation, differentiation, and recruitment, while also triggering systemic responses, such as the stimulation of liver hepatocytes to synthesize and release acute phase proteins^35–37^. Consequently, the levels of peripheral blood cytokines have emerged as a crucial diagnostic and prognostic biomarker for both systemic and localized inflammatory responses, offering potential applications in the assessment of therapeutic efficacy and the optimization of treatment strategies in various diseases, including sepsis, asthma, atherosclerosis and osteoarthritis^38–40^. Given that direct measurement of cytokines in affected tissues often necessitate tissue biopsy and cytokine detection in serum or plasma is challenging due to the short half-lives of many cytokines, their binding to soluble receptors or carrier proteins and the analytical difficulties originating from highly abundant plasma proteins, the measurement of cytokine production by peripheral blood mononuclear cells (PBMCs) has become a widely adopted approach for assessing inflammatory responses^41–51^. PBMCs, a readily accessible blood cell fraction comprised predominantly of lymphocytes and monocytes, play a crucial role in in mediating both innate and adaptive immune responses, regulating inflammation and maintaining immune homeostasis^41–52^. They act as sentinel cells, providing a real-time reflection of the cellular and humoral immune status state of the entire body^46,53–55^. Notably, the proteome of PBMCs has been demonstrated to correlate with the presence and progression of various diseases, including chronic obstructive pulmonary disease, rheumatoid arthritis, leukemia, pancreatic cancer, metabolic syndrome and sepsis^41–52,56^. Therefore, PBMCs are extensively employed as ex vivo cellular model in immunological studies to investigate immune responses across diverse inflammatory conditions, analyze diagnostic and prognostic biomarkers, identify potential immunotherapy targets, and assess the efficacy of immunomodulatory therapies^47,50,57–59^.

While in vitro or ex vivo assays with human immune cells offer valuable insights into immune function, they are unable to fully replicate the complex cellular and molecular interactions involved in immune responses^47,50,57–59^. Recognizing this limitation, the One Health initiative emphasizes the importance of companion animals as models for human disease, aiming to bridge the gap between medical and veterinary research for the benefit of both^60^. This necessitates the utilization of animal models for specific indications, with sheep, due to their anatomical and physiological similarities to humans, including organ size and longevity, emerging as widely used biomedical models^61–63^. These similarities encompass various systems, such as the cardiovascular and musculoskeletal system, where sheep and humans share characteristics in valve anatomy, heart rate, blood pressure, aorta size, hemodynamic flow parameters, weight, mechanical properties, joint structure, and bone architecture and remodeling processes^64–72^. Immunologically, both species exhibit cell-mediated and antibody-mediated responses to pathogens and antigens, and possess analogous immune organ structures^62,66,67^. Sheep respond to LPS challenges at doses comparable to human levels and utilize similar signaling pathways to activate immune responses^66–70^. Additionally, unlike small rodents, sheep exhibit population diversity through outbreeding and have a well-developed peripheral immune system by the time of birth^66–70^. Studies in sheep have significantly contributed to our understanding of the ontogeny and organization of the mammalian immune system^62,63^. However, the definitive establishment of the validity of sheep as animal models for immune and inflammatory diseases remains an ongoing pursuit. To validate sheep as models for the human immune system and inflammatory response, a comprehensive characterization of the cellular composition of sheep PBMCs and their immunological responses is imperative, which to date has been impeded by the limited array of available immunological tools^63,69,73^. Leveraging the well-documented high functional conservation observed in homologous proteins across species, with human and sheep proteins sharing approximately 93% amino acid identity^63^, mass spectrometry-based proteomics can facilitate immunophenotyping of PBMCs and characterization of their functional states^74–77^. A cross-species analytic approach also enables the identification of evolutionarily conserved hub proteins and pathways^78–88^, which, in turn, can inform the development of effective therapeutic strategies. Given that drug targets exhibit higher inter-species conservation than other genes and proteins, the co-occurrence of differential regulation in multiple species can be exploited for the identification and prioritization of therapeutic targets^78–88^.

Therefore, this cross-species comparative proteomics study aims to assess the suitability of the ovine model for replicating human immune signatures and inflammatory pathways by (1) establishing a protocol to isolate ovine PBMCs with cell ratios that closely resemble those found in hPBMCs to ensure accurate and meaningful comparisons in downstream analyses of ovine and human inflammatory responses, (2) identifying cell surface markers for ovine PBMCs using mass spectrometry based on human orthologs to facilitate comparison of PBMC composition and immunophenotype between studies, and (3) comparing the proteomic response of ovine and human PBMCs to inflammation integrating signals not only across orthologous individual molecules (proteins) but also at the level of functional sets, complexes, and pathways, where higher conservation is both expected and functionally more relevant.

## Materials and methods

### Sample collection and ethics approval

This study was carried out using peripheral blood obtained by venipuncture from the jugular vein of six healthy adult, 3–4-year-old Merino ewes, with ethical approval by the institutional ethics and animal welfare committee and the national authority (license BMWF-68.205/0116-V/3b/2018). All methods were performed in accordance with the relevant guidelines and regulations implemented at the University of Veterinary Medicine Vienna, the Institutional Ethics Committee (“Ethics and Animal Welfare Committee”) of the University of Veterinary Medicine Vienna.

All sheep included in the study were in a similar reproductive period (nongravid seasonal polyestrous) to ensure consistency in physiological conditions. They were confirmed to be systemically healthy by physical examination and the absence of hematologic abnormalities on complete blood count (CBC). Samples for CBC were subjected to routine blood cytometry performed by the University´s certified diagnostic laboratory within less than 3 h of collection. For oPBMCs isolation, 50 ml of venous whole blood were aseptically collected from the jugular vein into a heparinized (Gilvasan, 5000 IU/ml, 1 ml of Heparin/10 ml of blood) 100-mL syringe through a 23-gauge butterfly catheter. All samples were transferred to the lab and processed immediately after blood collection.

### Ovine PBMCs isolation

**Figure 1:**
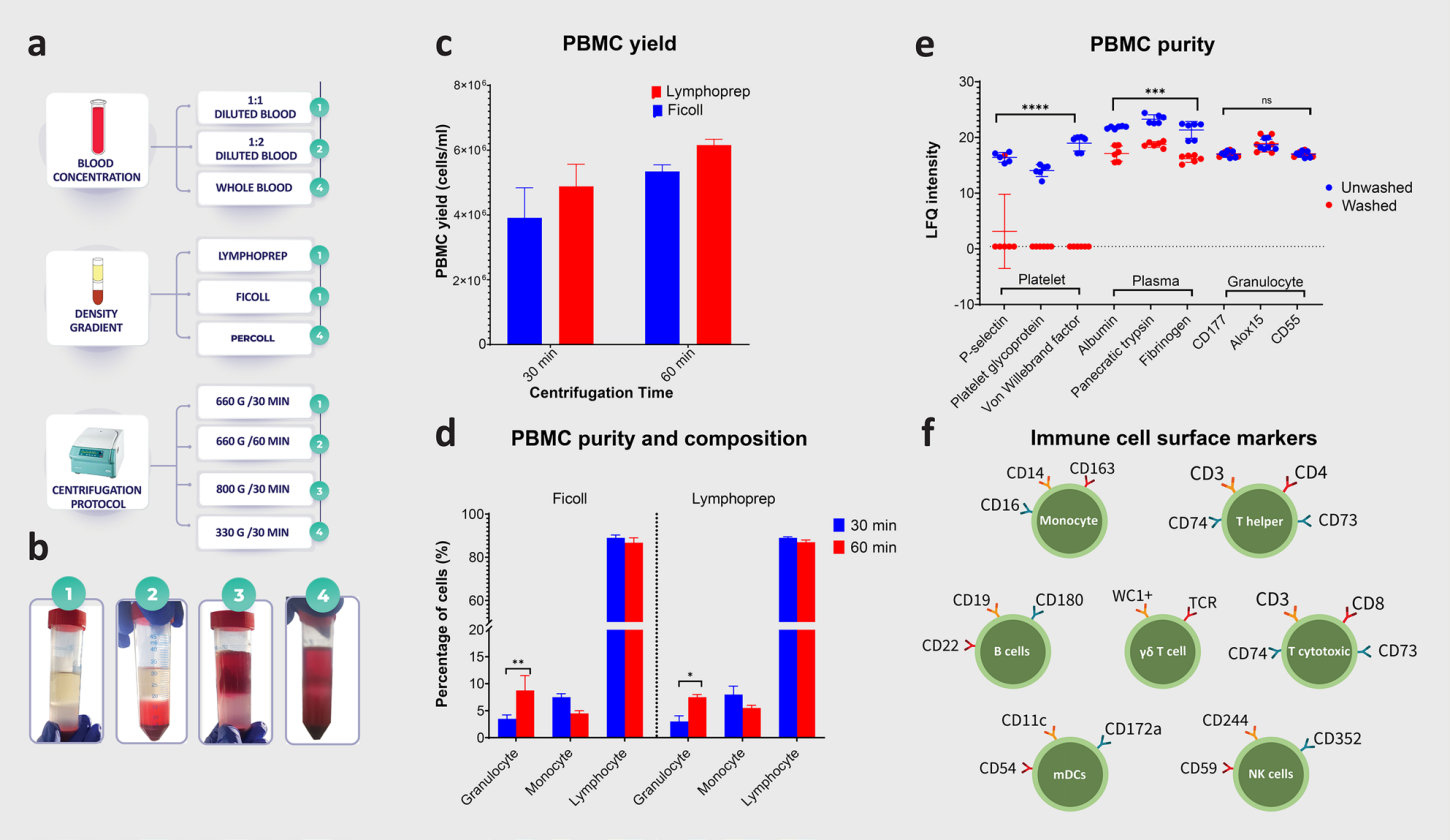
Optimization of the ovine peripheral blood mononuclear cell (oPBMC) isolation protocol. (**a**) Optimization of the blood dilution, density gradient and centrifugation parameters based on (**b**) PBMC separation quality. Selection of the density gradient based on (**c**) PBMC yield and (**d**) PBMC purity and composition. (**e**) Mass Spectrometry assessment of purity based on granulocyte-specific cluster of differentiation (CD) antigens and specific proteins associated with platelets and plasma. (**f**) Mass Spectrometry-based identification of orthologous CD antigens, indicating the presence of seven immune cell subsets.

The protocol for isolating oPBMCs was optimized, considering a diverse array of published technical parameters^89–102^ and accounting for the differences in the physical properties of ovine and human blood^103,104^. The optimization process included three primary variables: the dilution of blood samples, the selection of an appropriate density gradient medium, and the precise settings for centrifugation, encompassing both force and duration (Fig. 1a).

To determine the optimal ratio for blood dilution, anti-coagulated whole blood (n = 3 donors) was used either undiluted or diluted in a 1:1 or 1:2 ratio with complete RPMI1640 medium (Gibco, Life Technologies, Austria). This medium was supplemented with 10% heat-inactivated fetal calf serum (Gibco, Life Technologies, Austria), and 1% Penicillin, Streptomycin, and Amphotericin (Sigma-Aldrich, Germany,complete medium). The processed blood samples were then layered over three different density gradient media: Ficoll-Paque PREMIUM® (1.077 g/ml gradient, Cytiva, Sweden), Percoll® (1.130 g/ml gradient, GE Healthcare Bioscience, Sweden), and Lymphoprep® (1.077 g/ml, STEMCELL Technologies, Germany). These samples underwent centrifugation at three different centrifugation forces: 300×*g*, 660×*g*, and 800×*g*, each for a duration of 30 or 60 min (min), at 21 °C, and without brakes. The result of these experiments was ranked according to the quality of separation and perturbation of the different layers (Erythrocyte/Granulocyte layer, density gradient medium, PBMC layer, Plasma layer) (Fig. 1b).

Subsequently, the two density gradient media (Ficoll 1.077 g/ml versus Lymphoprep 1.077 g/ml) and centrifugation times (660×*g*/30 min versus 660×*g*/60 min), that achieved the best separation quality, were selected for further optimization, aiming to identify the protocol yielding the highest PBMC count with minimal granulocyte contamination. To this end, PBMCs were collected from the medium-plasma interface using a sterile pipet into a 50 ml conical tube and washed once at 540×*g* for 10 min at 21 °C using 20 ml of PBS without calcium and magnesium (PBS−/−). Then, 5 ml erythrocyte lysis buffer, composed of 154 mM ammonium chloride, 10 mM potassium hydrogencarbonate, and 0.1 mM Ethylenediaminetetraacetic acid), was added to the cell pellet. The tube was gently shaken to facilitate dissolution of the pellet, incubated for 5 min on ice, then mixed with 15 mL of PBS−/−, and centrifuged at 450×*g* for 5 min at 21 °C. Finally, the supernatant was discarded, and the cell pellet was washed twice using 10 ml of washing solution (PBS−/− with 2% FCS) and centrifugation at 440×*g* for 5 min at 21 °C. After the final wash and removal of the supernatant, the PBMCs pellet was processed for further analysis.

The yield of PBMCs was quantified by counting live cells per unit volume, determined by microscopic enumeration using a Neubauer hemocytometer. Cell viability was defined as the proportion of live cells in a population, assessed by their ability to exclude Trypan blue dye. The composition and purity of PBMCs were quantified using the ADVIA 2120i Hematology System™ Automated Cell Counter (Siemens, Germany). The composition of PBMCs was determined by calculating the ratio of isolated lymphocytes and monocytes to the total number of isolated PBMCs population (no monocytes/no PBMCs and no lymphocyte /no PBMCs), with the results expressed as percentage. The purity was determined as the percentage of PBMCs in the total isolated leukocyte population, with a specific emphasis on assessing contamination with other cell types such as granulocytes and erythrocytes. To ensure the suitability of the isolated PBMCs for downstream applications, stringent criteria were set, demanding a minimum viability of 95% and a purity exceeding 95%^105,106^.

### Proteomic phenotypic characterization of isolated oPBMCs

Due to the limited availability of ovine-specific antibodies essential for immunophenotyping techniques such as flow cytometry analyses^63,73^, oPBMCs were phenotypically characterized using MS-based proteomic analyses of lineage specific surface markers. The composition of PBMCs was determined based on the expression of cell type markers, while their purity was determined based on the presence or absence of granulocyte-specific CD antigens and specific proteins associated with platelets and plasma.

The PBMCs pellets were resuspended in serum-free medium (RPMI 1640 medium supplemented with 1% Penicillin, Streptomycin, and Amphotericin (Sigma-Aldrich, Germany) at a concentration of 4 × 10^6^ cells/ml. The cell suspension was plated into a T-25 flask (Greiner Bio-One, Kremsmünster, Austria) at a seeding density of 0.6 × 10^6^ cells per flask and incubated for 3 h at 37 °C in a humidified 5% CO2 incubator.

After the incubation time of the PBMCs, the conditioned medium was harvested into a 15 ml falcon tube, leaving approximately 1 ml medium in the culture flask. The adherent cells remaining in the flask were then gently detached using a cell scraper and combined with the previously transferred conditioned medium in the falcon tube. The cell suspension was centrifuged at 540×*g* for 5 min at 4 °C to pellet the cells and separate the supernatant. The supernatant was transferred into a new 15 ml falcon tube, centrifuged at 2000×*g* for 10 min at 4 °C and filtered through a 0.2 μm filter to remove potential remaining cells and cell debris. The filtered secretome was precipitated on ice cold 99.6% ethanol and stored at − 20 °C until further processing for isolation of secreted proteins.

The cell pellet obtained from the initial centrifugation was washed twice with 5 ml PBS−/− and centrifugation at 540×*g* for 5 min at 4 °C. Following the removal of the final wash solution, 200 μl of Sodium deoxycholate lysis bufferer (SDC) (4% sodium deoxycholate, 100 mM Tris HCl pH 8.5) was added to the cell pellet. The mixture was then heated at 95 °C in the water bath for 5 min to ensure complete lysis of the cells. The lysate was subsequently stored at − 80 °C until further proteomic processing for isolation cell lysate proteins.

The secretome and cell lysates obtained from unwashed PBMCs were used as control samples for the evaluation of cell purity. These cells were directly plated after the RBC lysis step as donor-matched control for each PBMC sample, bypassing the final two washing cycles, and were then designated for subsequent culture and analysis via mass spectrometry to measure cell type specific CD markers and specific proteins associated with platelets and plasma.

### Inflammatory stimulation oPBMCs

For a standardized assessment of inflammatory responses between ovine and human PBMCs, we adopted an inflammation induction protocol in oPBMCs consistent with the approach used for hPBMCs we previously described^107^.

In brief, isolated oPBMCs (n = 3 biological replicates (3 donors), 3 technical replicates/donor/experimental group) were resuspended to a final concentration of 4 × 10^6^ cells/ml in the complete RPMI 1640 medium supplemented with 1 μg/ml of lipopolysaccharide (LPS, Sigma-Aldrich, Merck, Darmstadt, Germany) in combination with 5 µg/ml of Phytohaemagglutinin (PHA, Sigma-Aldrich). The cell suspension was then seeded into a T-25 flask (Greiner Bio-One, Kremsmünster, Austria) at a density of 0.6 × 10^6^ cells per flask and incubated at 37 °C in 5% CO2 for 6 h. PBMCs cultured in complete RPMI 1640 medium without LPS or PHA served as healthy control samples. Following the 6-h period of inflammatory stimulation, the culture medium was changed to serum-free RPMI medium and further incubated at 37 °C in 5% CO2 for 3 h. Finally, both the secretome and cells were harvested for mass spectrometry analyses, as detailed previously in “Proteomic phenotypic characterization of isolated oPBMCs” section.

### Shotgun proteomics by LC–MS/MS

A quantitative LC–MS/MS of both the oPBMCs cell lysate and secretome of the washed versus unwashed PBMCs, as well as stimulated versus untreated PBMCs in sheep, was carried out.

#### Sample preparation

Proteomic samples were prepared using a modified version of a previously described protocol^108^ and employing an adapted version of the EasyPhos platform^109^. PBMC cell pellets were thawed, and further lysed using the S220 Focused-ultrasonicator (Covaris, LLC., Woburn, MA, USA). The precipitated secretome proteins were centrifuged at 5000×*g* for 30 min at 4 °C and the resulting protein pellet was solubilized in SDC buffer. Protein concentrations were determined via bicinchoninic acid assay (BCA)-assay. Protein (20 µg/sample) was reduced and alkylated with tris(2-carboxyethyl) phosphine (TCEP) and 2-chloroacetamide (2-CAM) for 5 min at 45 °C, followed by 18 h digestion with Trypsin/Lys-C (1:100 enzyme-to-substrate ratio) at 37 °C, and dried in a vacuum concentrator. Then, the samples were reconstituted in styrenedivinylbenzene-reverse phase sulfonate (SDB-RPS) loading buffer (99% iPrOH, 1% TFA) and desalted via SDB-RPS StageTips. Desalted global proteome samples were reconstituted in 5 µl formic acid (30%) containing synthetic standard peptides at 10 fmol and diluted with 40 µl loading solvent (98% H2O, 2% ACN, 0.05% TFA).

#### LC–MS/MS analysis

LC–MS/MS analyses were performed employing a timsTOF Pro mass spectrometer (Bruker Daltonics, Bremen, Germany) hyphenated with a Dionex UltiMateTM 3000 RSLCnano system (Thermo Scientific, Bremen, Germany). Samples were analyzed in data-dependent acquisition mode by label free quantification (LFQ) shotgun proteomics similarly to a recently published method^108^. The injection volume was 2 µl for cell lysates and 5 µl for secretomes, respectively. Samples were loaded on an AcclaimTMPepMapTM C18 HPLC pre-column (2 cm × 100 µm, 100 Å, Thermo Fisher Scientific™, Vienna, Austria) at a flow rate of 10 µl min-1 MS loading buffer. After trapping, peptides were eluted at a flow rate of 300 nl min-1 and separated on an Aurora series CSI UHPLC emitter column (25 cm × 75 µm, 1.6 µm C18, Ionopticks, Fitzroy, Australia) applying a gradient of 8–40% mobile phase B (79.9% ACN, 20% H2O, 0.1% FA) in mobile phase A (99.9% H2O, 0.1% FA) over 85 min.

#### LC–MS/MS data analyses

Protein identification was performed via MaxQuant^110^ (version 1.6.17.0) employing the Andromeda search engine against the UniProt Database^111^ (version 11/2021, 20′ 375 entries). Search parameters were set as previously described^108^. A mass tolerance of 20 ppm for MS spectra and 40 ppm for MS/MS spectra, a PSM-, protein- and site-false discovery rate (FDR) of 0.01 and a maximum of two missed cleavages per peptide were allowed. Match-between-runs were enabled with a matching time window of 0.7 min and an alignment time window of 20 min. Oxidation of methionine and N-terminal protein acetylation were set as variable modifications. Carbamidomethylation of cysteine was set as fixed modification. Proteome data analysis was performed via Perseus (version 1.6.14.0). Proteins with at least 70% quantification rate in at least one group were considered for analysis.

### Bioinformatics and statistical analyses

#### Differentially abundant proteins

To compare the inflammatory responses and pathways between ovine and human PBMCs, proteomics data from oPBMCs were juxtaposed with that of hPBMCs, with both sets inflamed and analyzed through the same methodological approach^107^. The mass spectrometry proteomics data of hPBMCs were retrieved from the ProteomeXchange Consortium through the proteomics identification database (PRIDE) repository with the dataset identifier PXD001415 (10.6019/PXD001415).

A two-sided Student’s t test was performed to examine differences between the control group and activated group, and the difference in abundance level between the two groups was calculated. Proteins satisfying a false discovery rate (FDR) ≤ 0.05 (used as the threshold of the q-value) and fold change (FC) │ ≥ 2│were considered to be significantly different (differentially abundant proteins, DAPs).

#### Enrichment analysis

Gene Ontology (GO) and Kyoto Encyclopedia of Genes and Genomes (KEGG) pathway enrichment analyses of the DAPs were performed using the Search Tool for Retrieval of Interacting Genes/proteins (STRING) database (version 11.5, https://string-db.org/)^111–116^ with a cut-off p < 0.05. The DAPs were assigned to their corresponding Gene Ontology branches (Biological Process, Molecular Function, and Cellular Component) and KEGG pathways, employing a species-specific background dataset for accurate comparison. Interactions analyzed were strictly confined to those substantiated by experimental evidence.

#### Protein–protein-interaction network construction and module analysis

Protein–protein interaction (PPI) networks were constructed using STRING (version 11.5; https://string-db.org), applying active interaction sources supported by experiments and an interaction score ≥ 0.4^117^, to identify functional interactions of DAPs. The PPI Networks were visualized and analyzed using the Cytoscape software (version 3.9.1, www.cytoscape.org) and its Molecular Complex Detection (MCODE) and CytoHubba plugins^118–120^. MCODE was employed to identify the main clusters in the PPI networks applying a degree cutoff = 2, node score cutoff = 0.2, K-core = 2, max. depth = 100, and haircut cluster finding setting as visualization criteria^119^. Clusters with a score ≥ 5 were considered significant subnetworks. With these clusters as input, we used STRING again to construct the second PPI network for further comprehensive enrichment analysis.

CytoHubba was utilized to calculate and rank the node scores of DAPs within PPI networks based on three hub protein-based identification algorithms, including the degree of connectivity, Maximal Clique Centrality (MCC), and Maximum Neighborhood Component (MNC). The top 30 hub proteins identified by each algorithm were plotted using Venn diagrams to determine overlapping proteins. The top overlapping proteins within the main cluster were designated as hub proteins^120–123^.

#### Pathway analysis

Pathway analysis was executed for the whole data set between the compared groups using Ingenuity Pathway Analysis (IPA) (QIAGEN Bioinformatics)^124^. The proteomic data sets, which comprised UniProt identifiers, p-values, and fold changes of total identified proteins, were imported into Ingenuity Pathway Analysis (IPA) for core analysis. The core analysis was conducted with the setting of direct and indirect relationships between molecules based on experimentally observed data, considering data sources in human databases within the Ingenuity Knowledge Base. IPA predicted potential canonical pathways of the proteins in this study, which were classified as activated or inhibited based on the Z-score, a statistical result of differential protein expression based on fold changes. Visualizing differentially affected pathways under different conditions was completed using the comparison analysis feature in IPA and hierarchical clustering. Pathways with Z score ≥ 2.0 (absolute) and p < 0.05 in at least one of the conditions were considered significant and reserved for comparison. Terms were filtered with respect to functional plausibility.

#### Statistical analysis

Statistical analyses were conducted using GraphPad Prism software (version 8.4.3). Continuous variables were expressed as mean ± standard deviation (SD), and categorical variables were expressed as percentages. Before statistical analysis, we assessed the normality of the data using the Shapiro–Wilk test^125^. Since the p-values were greater than 0.05 (p ≥ 0.05), suggesting normal distribution of the data, we employed parametric tests for the analyses. The differences in PBMC yield, purity and composition between the various PBMC isolation protocols and in CD marker expression and ratio between control and activated hPBMCs and oPBMCs were analyzed using ANOVA with Sidak correction for multiple comparisons when applicable. A two-sided Student’s t test was performed to analyze differences between the unwashed PBMCs and washed PBMCs. A p-value < 0.05 was considered significant.

### Ethics approval

This study was carried out using peripheral blood obtained by venipuncture from the jugular vein of six healthy adult, 3–4-year-old ewes, with ethical approval by the institutional ethics and animal welfare committee and the national authority (license BMWF-68.205/0116-V/3b/2018) and in accordance with the ARRIVE guidelines. No human participants were involved in this study; human proteomics data were retrieved from the ProteomeXchange Consortium through the proteomics identification database (PRIDE)^167^ repository with the dataset identifier PXD001415 (10.6019/PXD001415).

## Results

### Ovine PBMCs isolation

The oPBMCs isolation protocol was optimized regarding key variables including blood dilution, density gradient medium, and centrifugation parameters such as force and duration. Isolation protocols with blood diluted at 1:1 or 1:2 ratios in complete RPMI medium, utilizing either Lymphoprep or Ficoll for density gradient separation, and centrifuging at 660×*g* for durations of 30 or 60 min, achieved optimal layer separation. These protocols delineated four distinct layers—erythrocyte/granulocyte, density gradient medium, PBMCs, and plasma—more effectively and without perturbation than methods using undiluted blood, Percoll as the separation medium, and centrifugation forces of 800×*g* or 330×*g* (Fig. 1b).

Comparison of the effect of different centrifugation durations (30 min vs 60 min at 660×*g*) and density gradients (Ficoll versus Lymphoprep) on oPBMC isolation quantity and quality, revealed a statistically significant effect of density gradient (F = 34.64, DFn = 1, DFd = 2, p = 0.0277) but not centrifugation time (F = 7.681, DFn = 1, DFd = 2, p = 0.1093) on PBMC yield with Lymphoprep providing a higher PBMC yield (Table 1, Fig. 1c). In contrast, the percentage of granulocyte contamination was statistically significantly lower following a centrifugation time of 30 versus 60 min ((F = 44.67, DFn = 1, DFd = 2, p = 0.0217) but did not differ between density gradients (F = 1.762, DFn = 1, DFd = 2, p = 0.3156) (Table 1, Fig. 1d). Equally, PBMC composition (%lymphocytes vs % monocytes) differed significantly only between the different centrifugation times (F = 42.91, DFn = 1, DFd = 4, p = 0.0028) but not between density gradients (F = 0.8183, DFn = 1, DFd = 4, p = 0.4168) (Table 1, Fig. 1d). Across all protocols, cell viability remained at 100%, as confirmed by trypan blue exclusion. Furthermore, erythrocyte contamination was minimal (0.01–0.02 × 10^6^ / µL) or completely absent.

**Table 1:** Comparative analysis of oPBMC isolation efficiency: impact of centrifugation duration and density gradient media on the cell number (mean ± s.d.) isolated per ml blood and the percentage of isolated PBMCs.

Cell type	Centrifugation time (min)	Density gradient	Cell number isolated/ml blood		Percentage of isolated PBMCs	
			Mean	S.D	Mean	S.D.
PBMC total	30	Ficoll	3.91E+06	9.25E+05	96.4	1.9
		Lymphoprep	4.88E+06	6.83E+05	97.0	2.1
	60	Ficoll	5.34E+06	2.04E+05	91.2	2.8
		Lymphoprep	6.16E+06	1.77E+05	92.5	1.5
Granulocyte	30	Ficoll	1.39E+05	3.76E+04	3.6	0.7
		Lymphoprep	1.40E+05	3.19E+04	3.0	1.1
	60	Ficoll	4.71E+05	1.64E+05	8.8	2.8
		Lymphoprep	4.62E+05	2.48E+04	7.5	0.5
Monocyte	30	Ficoll	2.84E+05	7.91E+04	7.2	0.6
		Lymphoprep	3.99E+05	1.29E+05	8.0	1.6
	60	Ficoll	2.40E+05	1.76E+04	4.5	0.5
		Lymphoprep	3.38E+05	2.60E+04	5.5	0.5
Lymphocyte	30	Ficoll	3.49E+06	8.21E+05	89.2	1.3
		Lymphoprep	4.34E+06	5.84E+05	89.0	0.5
	60	Ficoll	4.63E+06	6.17E+04	86.8	2.3
		Lymphoprep	5.36E+06	2.00E+05	87.0	1.0

Prioritizing first the purity and then the yield of the PBMCs, the isolation technique using blood diluted 1:1 with complete RPMI medium, Lymphoprep density gradient, centrifugation at 660×*g* for 30 min and erythrolysis followed by two washing steps, proved most effective and was thus used for all subsequent experiments.

### Proteomic validation of the purity of isolated oPBMCs

MS-based proteomic analysis enabled efficient monitoring of contaminants, namely platelets and plasma and granulocytes. Notably, the optimized isolation protocol significantly diminished the presence of platelets (F = 242.5, DFn = 1, DFd = 14, p < 0.0001) and plasma proteins (F = 471, DFn = 1, DFd = 15, p < 0.0001) in the purified PBMCs (Fig. 1e). Granulocyte contamination, evidenced by the detection of granulocyte-specific CD antigens (CD177, CD55, and Alox15) through MS-based proteomic analysis, was minimal in both washed and unwashed PBMCs (F = 0.637, DFn = 1, DFd = 15, p = 0.437, Fig. 1e). This was quantitatively confirmed using a hemocytometer, revealing a mean contamination rate of 3% ± 1.1%. Therefore, the overall purity of isolated PBMCs, assessed by MS-based proteomic and hemocytometer analyses, exceeded 95%, confirming the protocol's efficiency and reliability for various downstream applications.

### Proteomic phenotypic characterization of isolated oPBMCs

MS-based proteomic analysis successfully identified 32 orthologous CD antigens with hPBMCs, categorizing PBMCs into six different immune cell subsets, encompassing both lymphocyte and myeloid cell lineages. Specifically, these subsets included CD14^+^ CD16^+^ monocytes, CD3^+^CD4^+^ T cells, CD3^+^CD8^+^ T cells, CD19^+^CD22^+^ B cells, CD11c^+^ mDCs, and CD244^+^CD352^+^ NK cells (Table 2, Fig. 1f). In addition, the proteomic analysis revealed pan-leukocyte markers, including CD74 and CD37. However, uniquely in oPBMCs, our results identified a WC1^+^ γδ T cell subset characterized by T-cell receptor (TCR) gamma chain (W5Q8Z2) and WC1.1-like antigens (W5QFU9) (Fig. 1f), which were not detected in hPBMCs within our datasets.

**Table 2:** Comparison of CD marker expression between ovine and human control and activated PBMCs (*indicates p < 0.05).

PBMCs cell type	Gene name	Human accession no	Sheep accession no	Protein name	Human control vs activated		Sheep control vs activated		Human versus sheep control		Human vs sheep activated	
					Mean diff	Adj. p	Mean diff	Adj. p	Mean diff	Adj. p	Mean diff	Adj. p
T cell	CD3E	P07766	W5PGT2	T-cell surface glycoprotein CD3 epsilon chain	6.9	0.44	0.1	1.00	− 12.9	0.88	− 19.7	0.75
	CD3D	P04234	W5PHC2	T-cell surface glycoprotein CD3 delta chain	− 6.6	0.41	− 0.9	0.98	− 44.2	0.19	− 38.5	0.27
	CD3G	P09693	W5PHL4	T-cell surface glycoprotein CD3 gamma chain	9.2	0.14	− 2.0	0.86	− 33.0	0.12	− 44.2	0.04*
	CD3-ZETA	P20963	W5PR78	T-cell surface glycoprotein CD3 zeta chain	− 12.4	0.01*	− 0.7	0.92	− 30.1	0.19	− 18.4	0.50
	CD4	P01730	W5P8J5	T-cell surface glycoprotein CD4	3.2	0.73	− 4.4	0.56	9.5	0.38	2.0	0.96
	CD8A	P01732	W5QHT2	T-cell surface glycoprotein CD8 alpha chain	− 3.9	0.61	10.1	0.11	40.0	0.04*	54.0	0.01*
	CD6	P30203	W5Q3F8	T-cell differentiation antigen CD6	5.8	0.85	1.3	0.99	4.0	0.98	− 0.6	1.00
	CD5	P06127	W5Q3P2	T-cell surface glycoprotein CD5	0.8	0.97	0.8	0.97	− 23.8	0.50	− 23.8	0.50
	CD45	P08575	W5Q2E5	Receptor-type tyrosine-protein phosphatase C	6.7	0.76	0.0	> 0.99	− 7.9	0.98	− 14.5	0.94
	CD166	Q13740	W5QBM4	CD166 antigen	− 3.9	0.92	− 14.4	0.38	11.3	0.83	0.7	1.00
	CD50	P32942	W5Q306	Intercellular adhesion molecule 3	6.9	0.29	13.6	0.05	22.8	0.60	29.5	0.44
	CD99	P14209	W5PED4	CD99 antigen	0.4	1.00	− 8.7	0.51	− 38.0	0.00*	− 47.1	0.00*
Monocyte	CD14	P08571	W5QJA2	Monocyte differentiation antigen CD14	21.2	0.09	10.7	0.39	48.6	0.05*	38.1	0.12
	CD163	Q86VB7	W5NY01	Scavenger receptor cysteine-rich type 1 protein M130	19.7	0.15	7.0	0.70	19.2	0.23	6.5	0.82
	CD9	P21926	W5PFL8	CD9 antigen	4.5	0.81	7.2	0.60	− 26.6	0.55	− 24.0	0.61
	CD11b	P11215	W5PGV0	Integrin alpha-M	− 0.1	1.00	10.1	0.04*	1.6	1.00	11.8	0.89
	CD16a	P08637	W5PK31	Low affinity immunoglobulin gamma Fc region receptor III-A	20.2	0.29	12.2	0.58	14.3	0.82	6.3	0.96
	CD18	P05107	W5PS30	Integrin beta-2	0.0	> 0.99	6.9	0.40	3.8	0.99	10.6	0.91
B cell	CD19	P15391	W5NUF5	B-lymphocyte antigen CD19	− 3.9	0.89	− 24.2	0.09	17.3	0.25	− 2.9	0.95
	CD22	P20273	W5P3Y9	CD22 molecule	2.7	0.94	− 8.8	0.57	− 32.8	0.00*	− 44.4	0.00*
	CD180	Q99467	W5P7C7	CD180 antigen	− 3.7	0.91	− 3.6	0.92	3.4	0.97	3.6	0.97
Natural Killer	CD352	Q96DU3	W5PGE8	SLAM family member 6	− 3.8	0.93	− 3.3	0.94	− 26.5	0.23	− 26.1	0.24
	CD244	Q9BZW8	W5PGV6	Natural killer cell receptor 2B4	2.2	0.54	2.0	0.59	11.7	0.16	11.5	0.17
	CD59	P13987	W5Q927	CD59 glycoprotein	− 9.1	0.36	− 2.5	0.91	− 46.0	0.02*	− 39.4	0.05*
Dendritic cells	CD11c	P20702	W5PH85	Integrin alpha-X	8.1	0.28	− 5.9	0.47	27.7	0.67	13.7	0.90
	CD172a	P78324	W5PVB4	Tyrosine-protein phosphatase non-receptor type substrate 1	13.2	0.02*	− 7.0	0.11	− 4.5	0.53	− 24.7	0.00*
	CD54	P05362	W5Q263	Intercellular adhesion molecule 1	− 9.6	0.08	− 43.2	0.00*	51.5	0.00*	17.8	0.23
Leukocyte	CD74	P04233	W5PBE0	HLA class II histocompatibility antigen gamma chain	5.0	0.63	− 13.8	0.11	− 29.5	0.46	− 48.3	0.17
	CD37	P11049	W5PTI0	Leukocyte antigen CD37	27.5	0.11	− 3.6	0.94	− 17.9	0.31	− 49.0	0.01*
	CD47	Q08722	W5QC22	Leukocyte surface antigen CD47	− 4.3	0.39	− 3.1	0.59	17.3	0.56	18.6	0.51
	CD44	P16070	W5QBV7	CD44 antigen	− 3.9	0.78	− 4.4	0.73	16.5	0.73	15.9	0.74
	CD58	P19256	W5QG77	Lymphocyte function-associated antigen 3	0.0	> 0.99	− 4.4	0.79	− 65.0	< 0.00*	− 69.3	< 0.00*

Remarkably, the majority (21 of 32) of CD markers displayed conserved patterns of expression, exhibiting no significant differences between species and activation states (p > 0.05). In contrast, 8 (of 32) CD markers (CD8a, CD99, CD22, CD59, CD172a, CD54, CD37, and CD58), were significantly differentially expressed between humans and sheep, and 4 CD markers (CD3-Z, CD50, CD14, and CD54) were significantly influenced by activation status (p < 0.05), with one CD marker (CD54) being significantly affected by species and activation states (Table 2, Suppl. Table 1).

Analysis of the various immune cell surface marker ratios, including T-cells: monocytes (CD3:CD16, CD3:CD163), T-cells:B-cells (CD3:CD19), T-cells:natural killer cells (CD3:CD352), T-cells:dendritic cells (CD3:CD11c), and T-helper cells:T-cytotoxic cells (CD4:CD8) across ovine and human, in both healthy control and inflamed PBMCs, based on their specific CD marker expressions, revealed no significant effects of species or activation status on the overall ratio of immune cell surface markers (p > 0.05). However, differential trends in specific immune cell surface marker ratios under different conditions were observed. Notably, healthy oPBMCs exhibited a 2.09 to 3.53-fold increase in the ratio of T-cell surface markers to the other PBMC surface markers compared to healthy hPBMCs, except for CD3:CD352, which was higher in humans (Table 3, Suppl. Table 2). In inflamed PBMCs, ovine CD-marker ratios for T-cells: monocytes and T-helper-cells: T-cytotoxic-cells were 2.5 to 21.5-fold higher compared to hPBMCs, respectively, while the other ratios were similar across species. (Table 3, Suppl. Table 2). Comparing the control and activated groups of PBMCs, both oPBMCs and hPBMCs showed similar patterns of either increased (CD3:CD16, CD3:CD163) or decreased (CD3:CD19, CD3:CD352) ratios. However, CD4:CD8 increased and CD3:CD11c decreased in sheep, while remaining constant in humans (Table 3, Suppl. Table 2).

**Table 3:** Comparison of surface marker ratios across species and activation states.

PBMCs cell type	Item	T cell: monocyte	T cell: monocyte	T cell: DC	T cell: B cell	T cell: NK cell	Thelper: tcytotoxic
CD group		CD3:CD16	CD3:CD163	CD3E:CD11c	CD3:CD19	CD3:CD352	CD4:CD8
Human control	Fraction	1.85	2.13	1.00	1.90	4.02	0.81
	Ratio	1:0.54	1:0.47	1:1.00	1:0.53	1:0.25	1:1.24
Sheep control	Fraction	3.87	7.53	2.17	4.85	1.79	2.61
	Ratio	1:0.26	1:0.13	1:0.46	1:0.21	1:0.56	1:0.38
Human activated	Fraction	4.11	5.83	1.02	1.51	2.88	0.70
	Ratio	1:0.24	1:0.17	1:0.98	1:0.66	1:0.35	1:1.44
Sheep activated	Fraction	10.41	25.15	1.85	1.89	1.66	14.96
	Ratio	1:0.1	1:0.04	1:0.54	1:0.53	1:0.60	1:0.07
ANOVA species	F (Dfn, Dfd)	F (1, 4) = 0.01	F (1, 4) = 0.9	F (1, 4) = 4.92	F (1, 4) = 0.32	F (1, 4) = 0.05	F (1, 4) = 0.25
	p value	0.92	0.39	0.09	0.60	0.83	0.64
ANOVA activation status	F (Dfn, Dfd)	F (1, 4) = 0.02	F (1, 4) = 0.75	F (1, 4) = 2.56	F (1, 4) = 0.33	F (1, 4) = 0.06	F (1, 4) = 1.23
	p value	0.89	0.44	0.18	0.60	0.82	0.33

### Proteome profiling of inflammatory stimulated ovine and human PBMCs

#### Mass spectrometry (MS)-based profiling

Upon inflammatory activation of PBMCs, the MS-based proteomic analyses profiled 4217 proteins in the whole cell lysates of oPBMCs and 4574 in hPBMCs, alongside 1913 proteins in the secretome of oPBMCs and 1375 in hPBMCs. This profiling was conducted after applying stringent filters for high confidence (FDR < 0.01 at both peptide and protein levels) and reproducibility, ensuring each protein was positively identified in at least 70% of the samples from one sample group. The comparative proteomic profiling of hPBMCs and oPBMCs demonstrated a notable interspecies overlap. Specifically, 47.8% of the proteins identified in the cell lysate (equivalent to 2790 proteins) and 32.8% of the secretome (comprising 988 proteins) were shared across both species.

#### Shared and species-specific differentially abundant proteins

In oPBMCs, 71 proteins and in hPBMCs, 176 proteins were differentially abundant (│FC│ ≥ 2, p < 0.05). Among the 71 DAPs identified in oPBMCs, 59 were upregulated (52 in whole cell lysate, 5 in secretome and 2 in both), while 12 were downregulated (11 in whole cell lysate and 1 in secretome) (Suppl. Table 3). In hPBMCs, out of 176 DAPs, 113 were upregulated (76 in whole cell lysate, 26 in secretome and 11 in both), whereas 63 were down-regulated proteins (51 in proteome, 11 in secretome and 1 in both) (Suppl. Table 4).

**Figure 2:**
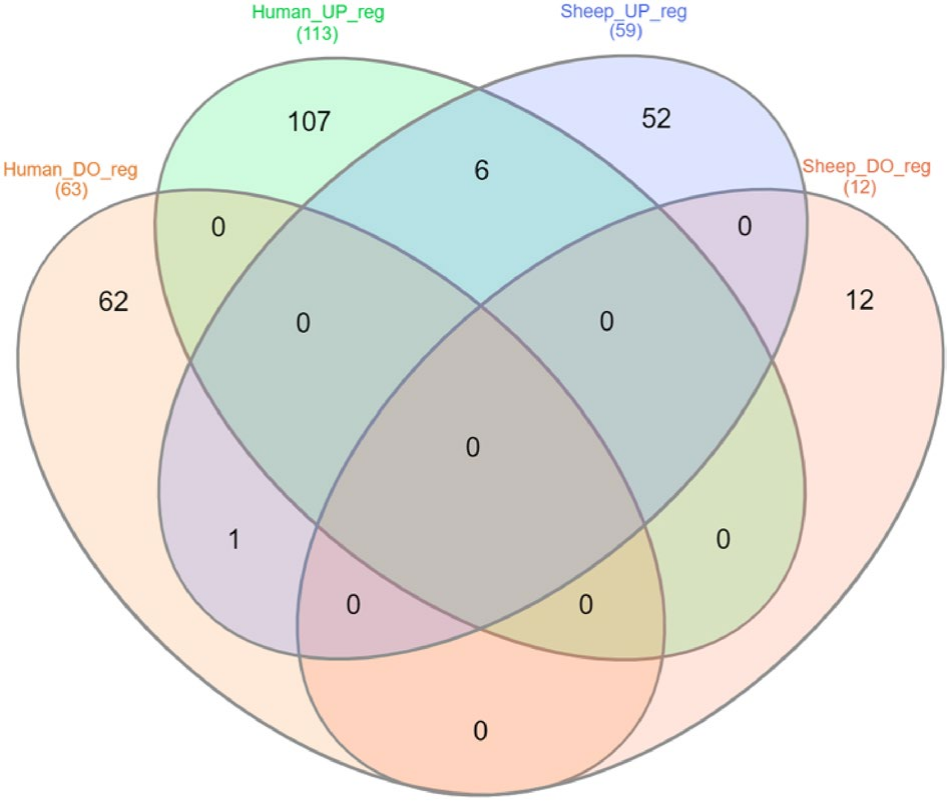
Comparative Venn analysis of differential abundant proteins (DAPs) in ovine vs. human peripheral blood mononuclear cells (PBMCs). Venn analysis of DAPs (fold change │≥ 2│, p < 0.05) that were upregulated (UP_reg) or downregulated (DO_reg) in response to inflammation in ovine and human PBMCs, yielded 6 overlapping upregulated DAPs (IL1B, IFIH1, CCL4, ISG20, IL1RN, and APOBEC3A) and 1 overlapping downregulated DAP (PDCD11), while the remainder of the DAPs were species-specific.

Venn analysis, capturing the overlap between 176 and 71 DAPs in human and sheep PBMCs, identified 7 overlapped DAPs (IL1B, IFIH1, CCL4, ISG20, IL1RN, APOBEC3A, and PDCD11), which were simultaneously associated with human and sheep activated PBMCs, as well as 169 human-specific DAPs (107 upregulated and 62 downregulated), and 64 sheep-specific DAPs (52 upregulated and 12 downregulated) (Fig. 2). The top 10 DAPs of activated PBMCs in humans and sheep are listed in Table 4.

**Table 4:** Top 10 differentially abundant proteins in activated vs. control PBMCs in sheep and human (Fold Change│ ≥ 2│, p < 0.05).

Species	Gene name	Accession no.	Protein name	p-value	Fold CHANGE	Subcellular location
Ovine	STAT3	W5NTT2	Signal transducer and activator of transcription	1.98E−02	2.14	Cell lysate
	**IL1B**	M4WG34	Interleukin-1 beta	1.33E−03	2.76	Secretome
				5.71E−03	2.71	Cell lysate
	IRF4	W5P0Y0	Interferon regulatory factor 4	2.30E−03	2.74	Cell lysate
	IFIH1	W5P825	RNA helicase	2.15E−02	2.70	Cell lysate
	IL17F	W5PXB0	Interleukin 17F	4.21E−03	2.69	Secretome
	TNFAIP3	W5NQZ0	Ubiquitinyl hydrolase 1	2.26E−02	2.65	Cell lysate
	IL17A	W5PWW6	Interleukin-17a	4.72E−03	2.60	Secretome
	SATB1	W5Q210	DNA-binding protein SATB	3.07E−03	2.60	Cell lysate
	STAT1	C8BKE1	Signal transducer and activator of transcription	5.42E−03	2.49	Cell lysate
	CCL4	W5P2A3	C–C motif chemokine	2.68E−02	2.21	Secretome
Human	**IL6**	P05231	Interleukin-6	1.65E−07	8.65	Secretome
				2.53E−04	4.45	Cell lysate
	**IL1B**	P01584	Interleukin-1 beta	3.71E−08	8.05	Secretome
				3.31E−05	4.20	Cell lysate
	**IFIT3**	O14879	Interferon-induced protein with tetratricopeptide repeats 3	5.30E−07	7.54	Cell lysate
				3.21E−03	3.99	Secretome
	CCL2	P13500	C–C motif chemokine 2	1.37E−06	7.52	Secretome
	**IL1A**	P01583	Interleukin-1 alpha	1.54E−06	7.26	Cell lysate
				5.14E−04	3.40	Secretome
	IFIH1	Q9BYX4	Interferon-induced helicase C domain-containing protein 1	2.49E−04	6.77	Cell lysate
	**CCL3**	P10147	C–C motif chemokine 3	3.56E−04	6.19	Secretome
				7.38E−04	4.08	Cell lysate
	CSPG2	P13611	Versican core protein	5.88E−07	− 5.64	Secretome
	**ISG15**	P05161	Ubiquitin-like protein ISG15	4.91E−04	5.33	Cell lysate
				1.27E−03	4.28	Secretome
	IFIT1	P09914	Interferon-induced protein with tetratricopeptide repeats 1	1.81E−04	4.95	Cell lysate

Proteins detected in both secretome and whole cell lysate datasets are highlighted in bold.

#### Enrichment analyses

DAPs of activated PBMCs were significantly enriched in 68 GO terms in sheep and 310 GO terms in humans (FDR < 0.05), of which 16 were shared between ovine and human PBMCs, 52 were ovine-specific and 294 human-specific (Suppl. Tables 5–7).

The shared biological process ontologies of DAPs included defense response, response to stress, immune response, defense response to virus, defense response to other organism, Inflammatory response, cellular response, interspecies interaction between organisms, response to other organism, innate immune response, and immune effector process. Molecular function ontology of DAPs was associated with protein binding, RNA helicase activity, and binding (Suppl. Table 7).

Enrichment analysis using KEGG pathways revealed 5 shared key pathway categories between ovine and human DAPs, including NF-kappa B signaling pathway, IL-17 signaling pathway, TNF signaling pathway, cytosolic DNA-sensing pathway, and cytokine-cytokine receptor interaction (FDR < 0.001, Suppl. Table 7).

#### Protein–protein-interaction network construction and module analysis

**Figure 3:**
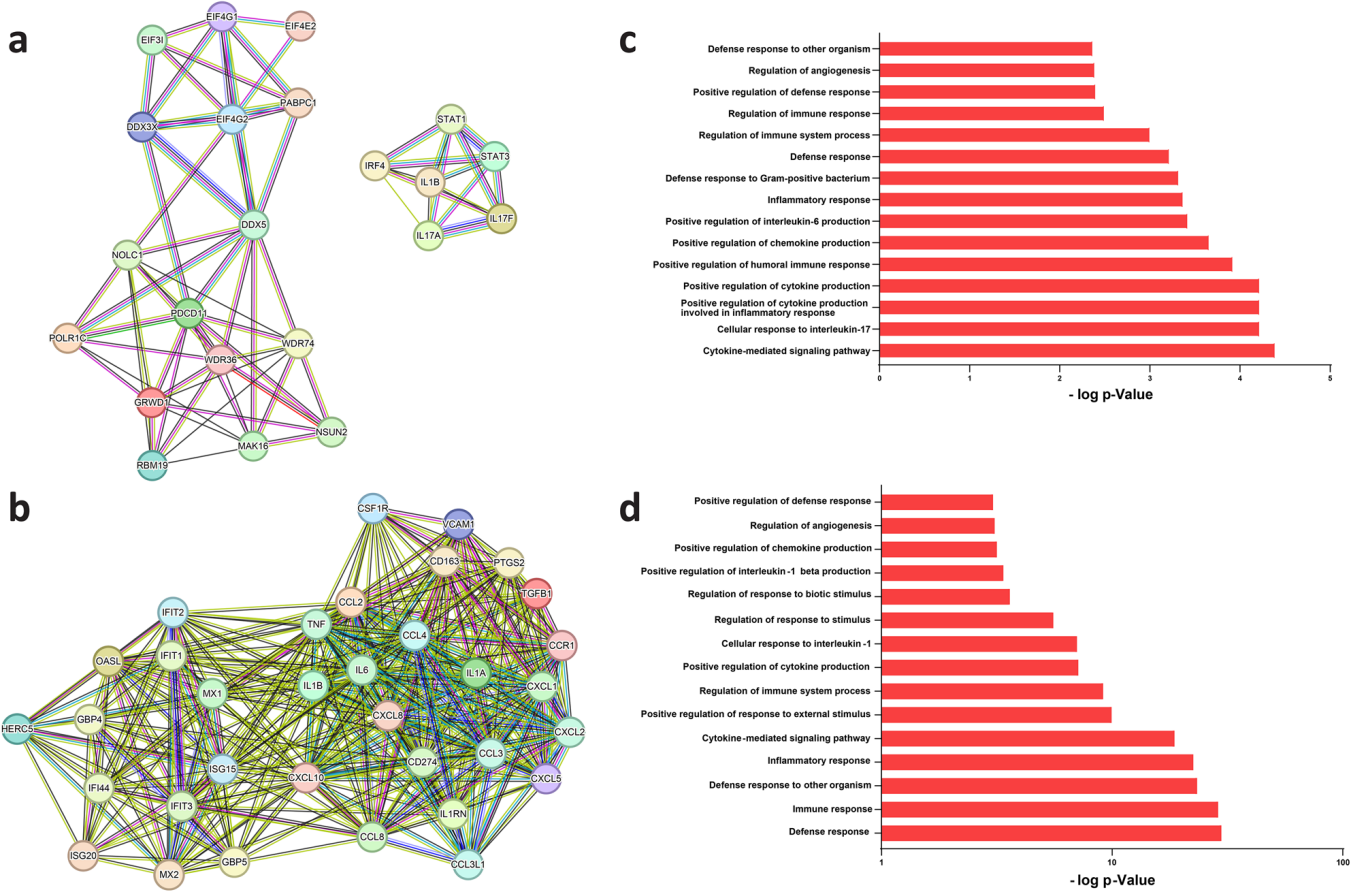
Comparative protein–protein interaction (PPI) networks and functional enrichment in ovine and human PBMCs, showing (**a**) the ovine main PPI cluster, (**b**) the human main PPI cluster, (**c**) functional enrichment of biological processes in ovine differentially abundant proteins (DAPs) and (**d**) functional enrichment of biological processes in human DAPs.

Protein–protein interaction (PPI) networks for DAPs of oPBMCs revealed 71 nodes, 219 edges vs. 73 expected edges (clustering coefficient: 0.475, enrichment p-value: 1.0E−16, average node degree: 6.17). In contrast, hPBMCs exhibited 168 nodes, 684 edges vs. 203 expected edges (clustering coefficient: 0.496, enrichment p-value: 1.0E−16, average node degree: 8.14). Furthermore, protein complex analysis of MCODE identified three clusters within the sheep PPI network, totally including 22 nodes and 74 edges (Fig. 3a, Suppl. Table 8), and one cluster within the human PPI network, totally including 34 nodes and 316 edges (Fig. 3b, Suppl. Table 8). The first and the second clusters in sheep were associated with RNA metabolism, and regulation of translational initiation (Suppl. Table 9). The third cluster in sheep, including six DAPs, and the primary cluster in human, including 34 DAPs, were predominantly associated with inflammatory responses. In the main cluster of DAPs 45 GO terms related to inflammatory biological processes were significantly enriched (FDR < 0.05) in sheep and 324 GO terms in humans (Suppl. Tables 9, 10). Within this set, 29 biological process terms were shared between the main clusters of sheep and human PBMCs (Suppl. Table 11), while 16 were specific to sheep and 295 were specific to humans. The top 15 biological process terms within the main cluster of PBMCs in humans and sheep are shown in Fig. 3c,d.

Venn analysis, capturing the overlap between three CytoHubba algorithms, identified 27 overlapped proteins for sheep and 28 for humans. Subsequently, the top overlapped proteins within the main cluster were designated as hub proteins. Within the main ovine cluster, the hub proteins were STAT1, IL1B, IRF4, STAT3, and IL17A, while in the main human cluster, they were CXCL10, CXCL8, IL1B, IL6, and TNF (Suppl. Tables 12–14). Remarkably, these hub proteins were identified as species-specific, with IL1B being the sole hub protein shared between activated PBMCs during the 6-h time course in both humans and sheep. Enrichment analysis confirmed the relevance of these hub proteins to inflammatory responses in both species.

#### Pathway analyses

Ingenuity pathway analysis of the differential proteomic expression profiles of hPBMCs secretome, oPBMCs secretome, hPBMCs whole cell lysate, and oPBMCs whole cell lysate, identified 17 canonical pathways that exhibited conserved activation/inhibition patterns across both species. Additionally, seven canonical pathways demonstrated divergent activation/inhibition patterns between both species (Table 5).

**Table 5:** Comparative analysis of canonical pathways in human and ovine PBMCs based on differential proteomic expression across secretome (*HSE* human PBMC secretome, *SSE* ovine PBMC secretome) and whole cell lysate (*HCL* human PBMC whole cell lysate, *SCL* ovine PBMC whole cell lysate) datasets.

Canonical pathways	HSE	SSE	HCL	SCL
Conserved activation patterns				
Role of hypercytokinemia/hyperchemokinemia in the pathogenesis of influenza	4.1	2.6	3.5	3.0
Interferon signaling	2.6	1.0	1.5	2.1
Autophagy	2.2	1.7	0.6	1.8
Inflammasome pathway	2.0	2.0	0.8	1.4
Acute phase response signaling	2.1	3.3	1.9	0.9
Ceramide signaling	1.2	2.1	1.5	1.2
NOD1/2 signaling pathway	2.8	1.0	0.7	0.6
Pathogen induced cytokine storm signaling pathway	2.3	1.8	0.9	0.8
Conserved inhibition patterns				
Serotonin receptor signaling	3.6	3.8	− 0.9	− 1.2
Phagosome formation	1.6	1.5	− 2.2	− 2.2
Integrin signaling	1.4	1.2	− 1.3	− 3.2
NF-κB activation by viruses	2.2	0.9	− 0.3	− 0.5
Role of PI3K/AKT signaling in the pathogenesis of influenza	2.0	1.1	− 0.7	− 0.7
ERK/MAPK signaling	2.3	2.3	− 0.3	− 2.4
IL-33 signaling pathway	2.7	1.5	− 0.4	− 0.5
CXCR4 signaling	2.5	1.4	− 1.2	− 0.5
IL-8 signaling	2.3	2.6	− 1.8	− 1.6
Divergent activation patterns				
HMGB1 signaling	4.6	1.7	0.8	− 0.4
Mitochondrial dysfunction	1.7	− 2.2	− 1.9	− 3.2
Glycolysis I	1.1	1.1	1.6	− 2.1
IL-6 signaling	2.4	2.3	1.5	− 1.3
p38 MAPK signaling	2.5	1.3	1.3	− 0.4
S100 family signaling pathway	2.6	1.8	0.9	− 1.1
IL-17 signaling	2.5	1.1	0.9	− 0.5

The top 5 cross-species conserved pathways that were activated in the secretome and whole cell lysate, were interferon signaling, inflammasome pathway, Pathogen Induced Cytokine Storm Signaling Pathway, NOD1/2 Signaling Pathway, and acute phase response signaling (Table 5). The top 5 cross-species pathways that were activated in the secretome and then inactivated at the level of the whole cell lysate in both species, were phagosome formation, CXCR4 signaling, IL-8 signaling, NF-κB Activation by viruses, and ERK/MAPK Signaling (Table 5).

The top 5 pathways with a species-specific activation pattern, that were activated in the secretome of both species but inactivated at the level of the whole cell lysate only in sheep, were IL-6 signaling, IL-17 signaling, p38 MAPK signaling , HMGB1 Signaling, and S100 Family Signaling Pathway (Table 5).

## Discussion

Sheep are commonly employed as a large animal model in immune-related studies^62,66,67,70^. However, inherent differences between human and sheep PBMCs may impact the translational relevance of research findings derived from sheep models. Therefore, in this cross-species comparative study, we examined the similarities and differences of the in vitro inflammatory response of ovine and human PBMCs by employing mass spectrometry to analyze the proteome of the PBMCs’ secretome and whole cell lysate.

The proteomic phenotyping of human and ovine PBMCs revealed 32 orthologous CD antigens with no significant difference in abundance levels between species. The surface markers indicated the presence of six distinct immune cell subsets in both human and ovine PBMCs, CD14^+^ CD16^+^monocytes, CD3^+^CD4^+^ T cells, CD3^+^CD8^+^ T cells, CD19^+^CD22^+^ B cells, CD11c^+^ mDCs, and CD244^+^CD352^+^ NK cells, consistent with established classifications^63,126–128^. However, ovine PMBCs also contained a unique WC1^+^ γδ T cell subset, not detected in hPBMCs. While the comparable abundance levels of immune cell subset markers indicate a similar composition of ovine and human PBMCs, establishing a crucial foundation for modeling inflammatory responses and interpreting subsequent proteomic shifts in both species, the presence of a unique T-cell subset introduces a potential confounding species-specific difference.

Comparative proteomic profiling of hPBMCs and oPBMCs revealed an overlap of approximately half (47.8%, 2790 proteins) of the entire cell lysate proteome, and one-third (32.8%, 988 proteins) of the secretome proteome between the two species. However, upon inflammatory stimulation, only seven differentially abundant proteins (IL1B, IFIH1, CCL4, ISG20, IL1RN, APOBEC3A, and PDCD11) were shared between sheep and humans, while 169 were specific to humans and 64 species-specific to sheep. This limited overlap, although consistent with comparable studies exploring proteome/transcriptome changes in human and mouse during Th17 cell differentiation^129,130^, is even more pronounced at the protein level of PBMCs in the current study. Two primary factors may contribute to this lack of overlap. First, considerable heterogeneity and compositional variations exist among circulating PBMCs in different species^50,131,132^. For instance, γδ T cells, a subset of lymphoid cells, typically constitute 0.5–10% of circulating T lymphocytes in adult humans^132,133^, while in adult sheep they represent up to 17%^131,133^ and in lambs 30–60%^134–137^. This heterogeneity may explain the successful identification of CD markers specific to the WC1^+^ γδ T cell subset in oPBMCs, a subset not detected in our hPBMCs samples. Second, inter-species differences in the cellular machinery arise from the intricate interplay between the conservation and diversification of regulatory mechanisms^87,138^. Therefore, incorporating signals not only at the level of orthologous individual molecules (proteins) but also within functional sets, complexes, and pathways is crucial when translating findings from ovine immunology to the human setting.

Using PPI networks and GO analyses, we identified 16 shared GO terms between both species with a strong representation of inflammatory-related processes. Enrichment analysis identified the major shared biological process “immune and inflammatory responses”, encompassing high-enrichment terms such as “leukocyte activation”, “leukocyte migration”, “leukocyte degranulation”, “leukocyte-mediated immunity”, “adaptive immune response”, “innate immune response”, and “cytokine production involved in immune response”^139^ that are associated with well-established consequences of inflammatory activation of PBMCs by LPS/PHA^55,107,140^.

Network analysis revealed five potential hub proteins in sheep and humans, primarily associated with inflammatory processes^55,98,101,141,142^. In sheep, the hub proteins included STAT1, IL1B, IRF4, STAT3, and IL17A, while in humans, they comprised CXCL10, CXCL8, IL1B, IL6, and TNF. Notably, IL1B, a potent pro-inflammatory cytokine with a pivotal role in orchestrating innate and adaptive immune responses^143,144^, emerged as the sole hub protein shared between both species, detected in the whole cell lysates and secretomes of PBMCs.

Considering that the secretome samples and the cell lysate samples were collected simultaneously, the obtained secretomes contain accumulated proteins synthesized and secreted over the incubation time (6 h in the current study), whereas the proteins obtained from the cell lysates give insight in the current cell status at the time point of collection. The current study utilized integrative global mass spectrometry-based proteomics analyses of both the secretome (extracellular) and whole cell lysate (intracellular) of PBMCs to assess of the pattern of activation/inhibition in shared signaling pathways and their underlying molecular mechanisms across both species and gain insight into the intricate regulatory mechanisms. In response to inflammatory stimulation, 17 canonical pathways, associated with the DAP of PBMCs of both species, exhibited consistent trends of activation/inhibition in both the secretome and the cell lysates (e.g., interferon signaling, inflammasome pathway, Pathogen Induced Cytokine Storm Signaling Pathway, acute phase response signaling, ERK/MAPK Signaling, CXCR4 Signaling, NF-κB Activation by Viruses, IL-33 Signaling, IL-8 Signaling, Integrin Signaling, etc.), emphasizing a high degree of conservation in immune and inflammatory responses across species. This observed conservation can be attributed to the substantial evolutionary conservation of inflammatory signaling and its transcriptional mechanisms in vertebrates^145,146^, despite variations in susceptibility and physiological differences between species^147–150^. For instance, the substantial homology between ovine and human Toll-like receptors (82–88% homology)^151,152^, as well as the close similarity in genomic responses and cardiopulmonary hemodynamics of sheep and humans challenged with lipopolysaccharide (LPS), further support the conservation^70,152–158^.

However, 7 divergent canonical pathways exhibited different trends of activation/inhibition in humans and sheep highlighting potential species-specific adaptive differences in the regulation of intracellular signaling pathways. Specifically, initial activation of “IL-6 signaling”, “HMGB1 signaling”, “p38 MAPK signaling”, “S100 family signaling pathway”, “IL-17 signaling”, “Mitochondrial Dysfunction”, and “Glycolysis I” was evident in the secretome of both species but rapid inhibition only in the whole cell lysate of sheep. These finding align with previous studies suggesting that differences in chemokine and cytokine expression and the response of various cell types to inflammatory cytokines across species might be related to species variability in regulation of inflammatory signaling pathways^70,159–161^. Inflammatory pathways are finely tuned by interconnected activating and inhibitory waves that delicately adjust the magnitude and duration of the inflammatory response over time to prevent tissue damage^162–165^. Thus, considering temporal changes in pathway regulation^163,166–168^ is crucial when translating pathways between sheep models and humans in future studies.

The lack of traditional immunochemical validation assays, primarily due to scarce sheep-specific antibodies, presents a methodological limitation of this study. However, Mass Spectrometry proteomics provides indirect validation by detecting proteomic patterns that are consistent with previously validated research^49,51–53,140,169–173^. Additionally, the MS-data provide a foundation for further refinement of the design of specific ovine antibodies for immuno-based analytical methods in future studies investigating immune repertoires in health and disease.

In conclusion, this cross-species comparative proteomics study sheds light on the intricate differences and shared aspects of the in vitro inflammatory response in ovine and human PBMCs, underscoring the importance of a judicious model selection to optimize the translatability of findings and uphold ethical standards in research. While significant similarities were found in conserved inflammatory pathways and biological processes, recognizing and addressing inherent species-specific differences is imperative when interpreting results of inflammation research results conducted in the ovine model. For inflammatory processes exhibiting divergence between the two species, the utilization of human-derived in vitro models or alternative animal models is recommended to optimize translational potential. Evidence-based selection of fit-for-purpose models ensures scientific quality and relevance of pre-clinical inflammation research while minimizing unnecessary animal use.

### Supplementary Information

Supplementary Table 1.

Supplementary Table 2.

Supplementary Table 3.

Supplementary Table 4.

Supplementary Table 5.

Supplementary Table 6.

Supplementary Table 7.

Supplementary Table 8.

Supplementary Table 9.

Supplementary Table 10.

Supplementary Table 11.

Supplementary Table 12.

Supplementary Table 13.

Supplementary Table 14.

## Data Availability

The datasets generated and analysed during the current study are included in this published article (and its Supplementary Information files) or available from the corresponding author on reasonable request.
